# Supplementary material for: Cranial MRI in Childhood Acute Leukemia during Treatment and Follow-Up Including the Impact of Intrathecal MTX—A Single-Center Study and Review of the Literature
Source: Cancers (Basel). 2022 Sep 26;14(19):4688. doi: 10.3390/cancers14194688 (PMC9563423; doi:10.3390/cancers14194688)
Supplement: Supplementary file 1 [file cancers-14-04688-s001.zip › cancers-1886635-supplementary.pdf]

cancers

MDPI

Supplement

Supplemental text S1: Details of the cMRI scans

Mainly axial T2 scans and flair- sequences were analyzed. To detect ischemia DWI sequence and for sinus vein thrombosis angiography were done. Most of the scans (90, from 2010 – 2017) were performed with a Magnetom Skyra 3.0 Tesla and the following parameters:

- SE-T2_tra: TR = 8610 ms, TE = 89 ms, FOV = 220 mm, SL = 4 mm, Pixel = 0.54 mm × 0.43 mm
- FLAIR_tra: TR = 10000 ms, TE = 81 ms, FOV = 220 mm, SL = 4 mm, Pixel = 0.88 mm × 0.57 mm
- Contrast enhanced_Angio_sag (in case of sinus vein thrombosis): TR = 3.1 ms, TE= 1.17 ms, FOV = 319 mm, SL = 0.8mm, Pixel = 0.83 mm × 0.83 mm

From 2007 – 2010 most scans (50) were performed with a Magnetom Sonata 1.5 Tesla and the following parameters:

- SE-T2_tra: TR = 4800 ms, TE = 108 ms, FOV = 220 mm, SL = 5 mm, Pixel = 0.86 mm × 0.86 mm
- FLAIR_tra: TR = 8200 ms, TE = 119 ms, FOV = 220 mm, SL = 4 mm, Pixel = 0.86 mm × 0.86 mm.

The remaining 30 scans were done with a Magnetom Expert 1.0 Tesla and a Magnetom Symphony TIM (SaTS) 1.5 Tesla, respectively.

Supplemental figure:

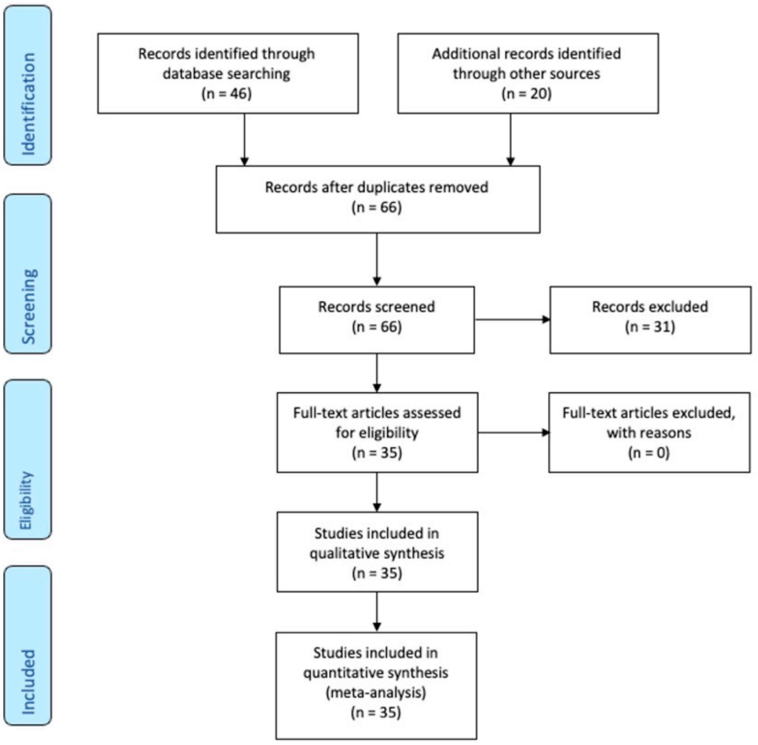

February 22, 2022

**#1**: child*[tiab] AND leukemia[tiab]	26,155 results
**#2:** neurotox*[tiab] OR (cns[tiab] OR "central nervous system"[tiab] OR brain[tiab] OR neuroradiolog*[tiab]) AND ("late effect*"[tiab] OR sequel*[tiab] OR consequenc*[tiab] OR "side effect*"[tiab]) AND ("MRI"[tiab] OR "magnetic resonance imag*"[tiab])	5,061 results
**#1 AND #2**	46 results

**Exclusion criteria**
Published before 1995.
No focus on ALL patients.
No focus on neuroradiological or neurocognitive findings related to leukemia treatment.
No focus on assessment with CT or MRI.
Focus on a small subgroup of patients (e.g., patients with stem cell transplantation)
Focus on secondary malignancies.
Case reports.

Supplemental figure S1: Search strategy, exclusion criteria and PRISMA flow diagram [36].

Supplemental tables

Supplemental table S1: Clinical features of patients at the time of diagnosis (P1) and during P2 and P3.

	ALL		AML		ALL + AML	
Clinical features at the time of diagnosis (P1)						
**Age [years]**						
Mean	8.8		9.6		8.9	
Median	7.1		10.5		7.1	
Range	0.2 – 23.9		0.9 – 23.0		0.23 – 23.9	
	**ALL**		**AML**		**ALL + AML**	
	**[n]**	**[%]**	**[n]**	**[%]**	**[n]**	**[%]**
**Diagnosis**	77	100	17	100	94	100
**Gender**						
Male	42	54.5	9	52.9	51	54.3
Female	35	45.5	8	47.1	43	45.7
**Risk group**						
IR	59	76.6	11	64.7	70	74.5
HR	18	23.4	6	35.3	24	25.5
**CNS positive at diagnosis**	6	7.8	1	5.9	7	7.4
Clinical features during P2 and P3						
**Cranial irradiation**	6	7.8	2	11.8	8	8.5
**Stem cell transplantation**	15	19.5	5	29.4	20	21.3
**Relapse**	13	16.9	5	29.4	18	19.1
**Death**	4	5.2	3	17.6	7	7.4

Supplemental table S2: Number and percentage of pathological findings during the different time periods. (CP = Cerebral pathomorphologies, WMC: white matter changes; ST: sinus vein thrombosis; BA: brain atrophy; HR = high risk; IR = intermediate risk; CIR = cranial irradiation) *7/8 patients receiving CIR were CNS positive. One patient with T-ALL was irradiated prophylactically.

		**General CP**		**WMC**		**ST**		**BA**		**Ischemia**	
	**Patients n**	Patients n / %	p – value	Patients n / %	p – value	Patients n / %	p – value	Patients n / %	p – value	Patients n / %	p – value
**ALL**	**77**	44 / 55.8	**p = 0.012**	15 / 19.5	p = 0.177	4 / 5.2	p = 0.909	33 / 42.9	**p = 0.016**	6 / 7.8	p = 0.786
**AML**	**17**	4 / 23.5		1 / 5.9		1 / 5.9		2 / 11.8		1 / 5.9	
**Male**	**51**	25 / 49.0	p = 0.666	7 / 13.7	p = 0.354	3 / 5.9	p = 0.791	18 / 35.3	p = 0.672	3 / 5.9	p = 0.529
**Female**	**43**	23 / 53.5		9 / 20.9		2 / 4.7		17 / 39.5		4 / 9.3	
**≤ 6 years**	**51**	31 / 60.8	**p = 0.040**	9 / 17.6	p = 0.860	3 / 5.9	p = 0.791	26 / 50.1	**p = 0.003**	4 / 7.8	p = 0.873
**> 6 years**	**43**	17 / 39.5		7 / 16.3		2 / 4.7		9 / 20.9		3 / 7.0	
**HR**	**24**	10/ 41.7	p = 0.286	6 / 25.0	p = 0.228	2 / 8.3	p = 0.446	6 / 25.0	p = 0.151	3 / 12.5	p = 0.275
**IR**	**70**	38 / 54.3		10 / 14.3		3 / 4.3		29 / 41.4		4 / 5.7	
**Relapse**	**17**	10 / 58.8	p = 0.479	5 / 29.4	p = 0.133	1 / 5.9	p = 0.191	6 / 35.3	p = 0.855	3 / 17.6	p = 0.077
**No relapse**	**77**	38 / 49.4		11 / 14.3		3 / 3.9		29 / 37.7		4 / 5.2	
**CIR***	**8**	6 / 75.0	p = 0.157	2 / 25.0	p = 0.530	1 / 12.5	p = 0.344	4 / 50.0	p = 0.435	1 / 12.5	p = 0.569
**No CIR**	**86**	42 / 48.8		14 / 16.3		4 / 4.7		31 / 36.0		6 / 7.0	
**All**	**94**	48 / 51.1		16 / 17.0		5 / 5.3		35 / 37.2		7 / 7.4	

Supplemental table S3: Literature overview. Summary of studies dealing with cerebral pathomorphologies detected in MRI or CCT in acute leukemias, children and adults. (Age and Follow-up time: (d: day; m: months; y: years); BBB: blood brain barrier; CNS: central nervous system; DTI: diffusion tensor imaging: DKI: diffusional kurtosis imaging; fMRI: functional MRI; HR: high risk; ith.: intrathecal; n.d.: not done; MRS: magnetic resonance spectroscopy; NT: neurotoxicity; PREST: posterior reversible leukoencephalopathy syndrome; SIADHS: syndrome of inappropriate antidiuretic hormone secretion; TIT: intrathecal triple drug (MTX, cytarabine, prednisone))

**White matter changes**									
**Diagnosis**	**Patients**	**Age**	**Follow-up time**	**Specific imaging findings**	**Neurological findings and symptoms**	**Percentage of pathologic MRIs**	**Explanation**	**Recommendations**	**References**
**ALL**	66	1 y – 9.99 y	≥ 2.6 y	leukoencephalopathy	neurocognitive deficits	68%	MTX	Restrictive use of MTX	[5]
	17 *(only CNS relapse)*	*diagnosis:* 0.3 y – 8.1 y relapse: 1.5 y – 11.3 y	median 4.0 y 0.1 y – 7.0 y	hyperdense regions	lower IQ-performance, disorder in fine motor skills, coordination	12.5%	cranial irradiation ith. MTX	Regular psychological and clinical follow up	[6]
	2444 *(review of 23 articles)*	0 y – 21 y	≥ 5 y	smaller hippocampus and impaired microstructural white matter integrity in frontal brain regions, impaired white matter integrity, altered functional connectivity	widespread reductions in brain activation during cognitive tasks, neurocognitive late effects	18.2% - 68% in 78.8% persistence	chemotherapy (damaging BBB, apoptosis of brain cells, DNA damage, oxidative stress, shorter telomere length, impaired neurogenesis)	exploiting multiple MRI techniques, monitoring of intracerebral changes throughout therapy and during long-term follow-up, longitudinal studies combining neuroimaging and neurocognitive outcome	[8]
	48	mean: 7 y 2 y – 15 y	mean: 26 m 9 d – 56 m	frontal and temporal leukoencephalopathy	no correlation to neurocognitive impairment	50% after cranial irradiation 66.6% after ith. MTX	cranial irradiation ith. MTX	further long-term studies	[10]
	38	median 4 y 1 y – 15 y	mean: 38 y 27 y – 46 y	microstructural damage in white matter, fornix, uncinate fasciculus, and ventral cingulum *(DTI, DKI)*	neurocognitive lower scores: vocabulary, memory, learning capacity, spatial ability, executive functions, and attention	100%	cranial irradiation ith. MTX	usage of DTI and DKI to evaluate integrity of white matter	[11]
	190	3.3 y – 10.8 y	≥ 5 years	leukoencephalopathy without cranial radiation: higher risk for reduced white matter integrity in frontal brain regions	leukoencephalopathy without cranial radiation: higher risk for long-term neurobehavioral problems	27% 78% have continuous leukoencephalopathy	ith. MTX; dexamethasone	cognitive and behavioral interventions	[12]
	15	1.6 y – 9.4 y	3.6 m – 6.8 m	white matter changes mainly frontal *(diffusion tensor imaging)*	n.d.	-	MTX	prospective long-term follow-up	[15]

	45	4 y – 17 y	6 – 12	MRS is more sensitive than MRI	no unusual neurological findings	11%	cranial irradiation MTX	none	[17]
	20	2.2 y – 13.7 y	16 y – 28 y	white and grey matter changes	n.d.	-	cranial irradiation	follow-up	[18]
	98 + 7 relapses	4 y – 16 y	9 d – 6 y	leukoencephalopathy calcifications	headache, seizures, change of mental status	6%	treatment, relapse, infections	early diagnosis studies to genetic polymorphism for risk factors	[19]
	1218	14 m – 227 m	1 d – 86 d	Leukoencephalopathy ± calcifications *(only checked in 95 symptomatic patients)*	seizures	7.8% acute neurotoxicity up to 77.1% MRI changes in symptomatic patients	intensification of i.v. MTX and TIT had 2- to 3-fold increase of acute NT and a 5-fold incidence of leukoencephalopathy	earlier leucovorin rescue long-term follow-up regular MRI/CT to find occult changes neuropsychological testing	[22]
	14	3 y – 16 y	up to 3 y	leukoencephalopathy, meningeal enhancement, lesion in ganglia and white matter	seizures, change of mental status	7/14	leukemia, treatment, infection	early diagnosis	[23]
	35	median: 69 m 14 m – 186 m	median: 7.7 y	pathological hyperintensity, calcifications	13/27: eye deviations 13/27: seizures 4/27: speech disorders headache	11%	cranial irradiation ith. MTX	Rapid diagnosis and treatment periodic neurocognitive testing CT and MRI not for subtle alterations	[34]
	25	6.9 y ± 3.0 y	6 y	leukoencephalopathy	no correlation	4%	cranial irradiation, HR	prospective studies	[41]
	129	0 y – 19 y	At the end of treatment	leukoencephalopathy more frequent in cases of lower age and higher cumulative IV-MTX doses; extend of leukoencephalopathy with i.v. MTX doses	no correlation	53%	low age i.v. MTX	follow-up of younger patients and those with i.v. MTX to understand the neurotoxic mechanisms of chemotherapy, its long-term neurological impact and how to minimize possible deficits	[42]
	19	mean: 5.7 3 y – 12 y	mean: 8.6 y 5 y – 15 y	leukoencephalopathy no association with age, radiotherapy dose, MTX-dosage, or CNS involvement	lower IQ 3 patients: abnormal auditory 5 patients: abnormal visual evoked potential	33.3%	cranial irradiation	prospective studies	[45]
	55	median: 3.5 y 1.1 y – 6.6 y	8.9 y – 13.1 y	*no cranial irradiation:* 6 patients with abnormalities *with cranial irradiation:*	poorer memory and fine-motor functioning outcome	*no irradiation* 38% *with irradiation:* 63%	cranial irradiation chemotherapy	rehabilitation of children with treatment-associated cognitive impairment is essential	[46]

				15 patients with abnormalities	no significant relationships between MRI outcome and test scores, school placement, or education level			follow-up	
**AML**	**No reports**								
**Sinus Thrombosis**									
**Diagnosis**	**Patients**	**Age**	**Follow-up time**	**Specific imaging findings**	**Neurological findings and symptoms**	**Percentage of pathologic MRIs**	**Explanation**	**Recommendations**	**References**
**ALL**	98	4y – 16y	9d – 6y	superior sagittal sinus	headache, seizures, change of mental status	2%	treatment, relapse, infections	early diagnosis studies to genetic polymorphism for risk factors	[19]
	14	3y – 16y	under treatment	superior sagittal sinus	seizures, hemiparesis	2/14	leukemia, treatment	early diagnosis	[23]
**AML**	5	3y – 16y	under treatment	sigmoid sinus	seizure, limb weakness	1/5	leukemia, treatment	early diagnosis	[23]
**Brain atrophy**									
**Diagnosis**	**Patients**	**Age**	**Follow-up time**	**Specific imaging findings**	**Neurological findings and symptoms**	**Percentage of pathologic MRIs**	**Explanation**	**Recommendations**	**References**
**ALL**	79	6.3 y – 21.7 y	30m – 75m	decreased volume of selected subcortical structures	full scale IQ, verbal learning, cognitive impairment	100%	cranial irradiation	further studies	[3]
	33	6.7 y – 19.9 y	4 y	decreased hippocampal and nucleus caudatus volume	lower IQ, poorer verbal abilities	-	cranial irradiation, high dose chemotherapy	further studies	[4]
	17 *(only CNS relapse)*	*diagnosis:* 0.3 y – 8.1 y relapse: 1.5 y – 11.3 y	median 4.0 y 0.1 y – 7.0 y	grey and white matter atrophy	lower IQ-performance, disorder in fine motor skills and coordination	56%	cranial irradiation ith. MTX	regular psychological and clinical follow up	[6]
	13	2.2 y – 13.7 y	16 y – 28 y	decrease of white and grey matter volume		-	cranial irradiation	follow-up	[18]
	20	Mean 14 y	14 y	volume loss of hippocampus, thalamus and temporal lobe white and grey matter changes	should be tested	-	cranial irradiation	prospective studies	[20]
	218	1 y – 18 y	5 y – 10 y	no volume changes but smaller hippocampi and	n.d.	-	dexamethasone	lower doses of dexamethasone for	[28]

				cerebelli in survivors, females risk factor				younger females, NMDA receptor antagonist avoid irradiation	
	25	6.9 y ± 3.0 y	6 y		no correlation	4%	cranial irradiation, HR	prospective studies	[41]
	67	mean: 3.8 y 1 y – 10 y	≥ 2 y mean: 8.1 y	widespread reductions in brain volume a subtle, global alteration in white matter microstructure *(altered diffusion)*	average IQ 95 compared to 110 in controls no significant correlation to imaging	6% less white matter 5% less gray matter	leukemia, treatment	further research needed to understand how these alterations emerge	[43]
	23	mean: 4.4 y 2,1 y – 8.4 y	≥ 2 y 3 y – 11 y	reduced white matter reduced gray matter temporal, occipital	more poorly in working memory and response inhibition correlations between working memory and volume of amygdale, thalamus, striatum, and corpus callosum	-	chemotherapy	large scale studies are needed to establish time-course of changes for understanding	[44]
**AML**	**No reports**								
**Ischemia**									
**Diagnosis**	**Patients**	**Age**	**Follow-up time**	**Specific imaging findings**	**Neurological findings and symptoms**	**Percentage of pathologic MRIs**	**Explanation**	**Recommendations**	**References**
**ALL**	25	6.9 y ± 3.0 y	6 y	old infarct and hemorrhage		4%	cranial irradiation, HR	prospective studies	[41]
**AML**	5	0.6y – 13y	under treatment	disseminated tiny lesions in thalamus and cerebral white matter	encephalopathy	1/5	leukemia, treatment, infection	early diagnosis	[23]
**Other findings or not further specified**									
**Diagnosis**	**Patients**	**Age**	**Follow-up time**	**Specific imaging findings**	**Neurological findings and symptoms**	**Percentage of pathologic MRIs**	**Explanation**	**Recommendations**	**References**
**ALL**	1378	1.0 y – 17.9 y	median: 33 d 10 d – 1254 d	PRES (n = 52)	late: epilepsy (n = 7), neurocognitive impairment (n = 7)	3.8% (52/1378)	older age, T-cell ALL, CNS positive, treatment	MRI imaging	[2]
	850	2 y – 11 y	17 d – 34 d	PRES (n = 12)	Seizures, visual disturbances, conscious disturbances, no effect on IQ and cognitive development	1.5% (13/850)	induction chemotherapy	MRI imaging	[9]

	26	2.3 y – 7.0 y	median: 35 y<br>32 y – 37 y	no altered fMRI activity	longer response times and reduced accuracy performance during cognitive interference processing	-	-	-	[13]
	256	median:<br>69 m<br>14 m – 186 m	during treatment in case of n s ymptoms (neurolog y)	PRES (n = 10), stroke (n = 5),	temporal lobe epilepsy (n = 2), high-dose methotrexate toxicity (n = 2), SIADH (n = 1), and other unclassified events (n = 7)<br>not correlated with imaging	11%	up to 9 years	early identification to prevent late effects	[16]
	98<br>+ 7<br>relapses	4 y – 16 y	9d – 6 y	hemorrhage<br>meningioma, osteoma<br>CNS lymphoma	palsy, hemiplegia, headache, ataxia	9%	treatment, relapse, infections	early diagnosis<br>studies to genetic polymorphism for risk factors	[19]
	122	mean: 5.3 y<br>1 m – 17 y	Mean: 90 m	high signal intensities<br>*(MRI only in patients with neurological symptoms, n=10)*	seizures, ataxia, flaccid paralysis	8.2% (10 patients) 8/10: MRI changes	TIT	further studies are needed	[21]
	14	3 y – 16 y	up to 3 y	hemorrhage, infarct, inflammation, infections, intracerebral tumor, CNS relapse	Seizures, mental changes, fever, hemiparesis, headache	4/14	leukemia, treatment, infection	early diagnosis	[23]
	25	6.9 y ± 3.0 y	6 y	bleeding	no correlation	4%	cranial irradiation, HR	avoid irradiation	[41]
	25	1 y – 11 y	0 d – 5 m	hemorrhage<br>leukemia infiltrations<br>aspergillosis	hemiplegia<br>headache<br>fever, sepsis	24%	treatment, relapse, infections	early diagnosis<br>studies to genetic polymorphism for risk factors	[19]
	5	0.6 y - 13 y	acute	<u>*acute:*</u> disseminated microinfarcts, infections, vasculopathy<br><u>*late:*</u> chordoma	seizures, paresis	3/5	leukemia, treatment, infection	early diagnosis	[23]
